# Supplementary material for: Galectin-3 Is Associated with Cardiac Fibrosis and an Increased Risk of Sudden Death
Source: Cells. 2023 Apr 23;12(9):1218. doi: 10.3390/cells12091218 (PMC10177039; doi:10.3390/cells12091218)
Supplement: Supplementary file 1 [file cells-12-01218-s001.zip › cells-2323064-supplementary.pdf]

Supplemental Tables

***Supplemental Table S1.* Socio-Demographic and baseline characteristics of the study subjects who died suddenly from cardiac or non-cardiac causes along with concomitant clinical variables and cardiac tissue morphometry**

**Categorical Variables**		**Non-cardiac cause of death (n= 54)**	**Cardiac cause of death (n=167)**	**Total (n=221)**	**P-value**
Race	Caucasian	35 (27%)	97 (73%)	132	0.496
	African American	16 (21%)	59 (79%)	75	
	Hispanic	3 (38%)	5 (63%)	8	
	Other	0 (0%)	3 (100%)	3	
ICD Present		0 (0%)	9 (100%)	9	0.054
Appropriate ICD shock		0 (0%)	4 (100%)	4	0.123
DM		12 (14%)	71 (86%)	83	0.028*
HTN		29 (19%)	124 (81%)	153	0.013*
Tobacco		21 (20%)	84 (80%)	105	0.244
Public Arrest		2 (11%)	17 (89%)	19	0.321
Home Arrest		13 (19%)	54 (81%)	67	0.498
Hospital Arrest		38 (29%)	94 (71%)	132	0.153
Rhythm Prior to SCA	Sinus rhythm	6 (50%)	6 (50%)	12	0.000*
	Ventricular Tachycardia	0 (0%)	15 (100%)	15	
	Ventricular Fibrillation	0 (0%)	45 (100%)	45	
	PEA	20 (33%)	41 (67%)	61	
	Asystole	7 (28%)	18 (72%)	25	
Atrial Fibrillation		11 (22%)	40 (78%)	51	0.353
Beta Blocker		16 (18%)	74 (82%)	90	0.07
ACEi/ARB		19 (22%)	66 (78%)	85	0.629
Statin		11 (13%)	74 (87%)	85	0.002*
Aspirin		9 (11%)	75 (89%)	84	0.000*
Diuretic		14 (18%)	65 (82%)	79	0.112

Results are expressed as absolute numbers (percentage). *, p-values of <0.05.
DM, diabetes mellitus; HTN, hypertension; ICD, implantable cardioverter defibrillator; SCA, sudden cardiac arrest; PEA, pulseless electrical activity; ACEi, angiotensin-converting enzyme inhibitor; ARB, angiotensin-receptor blocker.

***Supplemental Table S2*. Risk factors, clinical data, and significant laboratory values of the study subjects who died suddenly from cardiac or non-cardiac causes**

**Continuous Variables**	**Non cardiac cause of death (n= 54)**	**Cardiac cause of death (n=167)**	**Total (n=221)**	**P-value**
QRS	100 ±25.1	109.2 ±33	106.8 ±31.3	0.093
QTc	425.3 ±40.2	419.3 ±60.2	420.8 ±55.6	0.539
Potassium	4.6 ±0.9	4.5 ±1.0	4.5 ±1.0	0.316
Magnesium	2.2 ±0.6	3.5 ±13.5	3.1 ±11.4	0.541
Sodium	140.3 ±4.9	137.4 ±15.7	138.1 ±13.8	0.211
Hemoglobin	10 ±2.9	10.6 ±2.6	10.5 ±2.7	0.162
Cholesterol	178 ±29.2	156.7 ±57.7	160.3 ±54.3	0.262
Triglycerides	120.8 ±80.3	139.4 ±111.1	136.1 ±105.8	0.602
High Density Lipoprotein	46.5 ±19.6	37.2 ±14	38.8 ±15.4	0.081
Low Density Lipoprotein	108.1 ±28.7	97.2 ±43.6	99 ±41.4	0.451
Very Low Density Lipoprotein	25.5 ±19.1	27.8 ±22.4	27.5 ±21.8	0.800
Cholesterol/HDL	4.9 ±3.1	4.9 ±2.8	4.9 ±2.8	0.945
LDL/HDL ratio	3.3 ±2.5	3 ±2	3.1 ±2.1	0.709
BNP	6445.1 ±17385	3264.1 ±8568.5	3982.4 ±11142.4	0.252

Results in Supplemental 2 are expressed as mean ± SD, statistical significance was tested by student T-test or Chi-squared test, as appropriate.

HDL, High density lipoproteins; LDL, low density lipoprotein; BNP, brain-natriuretic peptide.
